# Genome Sequencing of *Hericium coralloides* by a Combination of PacBio RS II and Next-Generation Sequencing Platforms

**DOI:** 10.1155/2022/4017654

**Published:** 2022-01-31

**Authors:** Caixia Zhang, Lijun Xu, Jian Li, Jiansong Chen, Manjun Yang

**Affiliations:** ^1^Tibet Vocational Technical College, Lhasa, Xizang, 850000, China; ^2^Tibet University of Tibetan Medicine, Lhasa, Xizang, 850000, China; ^3^China Institute of Veterinary Drug Control, Beijing 100081, China; ^4^School of Life Sciences, Instrumental Analysis and Research Center, Sun Yat-sen University, Guangzhou 510006, China

## Abstract

The fruiting bodies or mycelia of *Hericium coralloides* (*H. coralloides*) contain many physiologically active compounds that are used to treat various diseases, including cardiovascular disorders and cancers. However, the genome of *H. coralloides* has not been sequenced, which hinders further investigations into aspects, such as bioactivity or evolutionary events. The present study is aimed at (i) performing *de novo* sequencing of the assembled genome; (ii) mapping the reads from PE400 DNA into the assembled genome; (iii) identifying the full length of all the repeated sequences; and (iv) annotating protein-coding genes using GO, eggNOG, and KEGG databases. The assembled genome comprised 5,59,05,675 bp, including 307 contigs. The mapping rate of reads obtained from PE400 DNA in the assembled genome was 92.46%. We identified 2,525 repeated sequences of 14,23,274 bp length. We predicted ncRNAs of 48,895 bp and 11,736 genes encoding proteins that were annotated in the GO, eggNOG, and KEGG databases. We are the first to sequence the entire *H. coralloides* genome (NCBI; Assembly: ASM367540v1), which will serve as a reference for studying the evolutionary diversification of edible and medicinal mushrooms and facilitate the application of bioactivity in *H. coralloides*.

## 1. Introduction

Wild edible mushrooms are extensively consumed owing to their unique and delicate flavors, abundant polysaccharides, proteins, fibers, and amino acids, and low lipid content that is good for low-calorie diets [1–3]. Except for the nutritional characteristics, mushrooms also contain rich bioactive compounds with medicinal properties, such with antimicrobial, antioxidant, lipid-lowering, and antitumor activity [4–6].

*Hericium coralloides* (1794) is an edible and medicinal mushroom species. The members of genus *Hericium* produce fleshy, whitish basidiomata, and their fruiting bodies or mycelia have been widely applied in traditional Chinese medicine (TCM) to treat various diseases, including cardiovascular disorders, cancer [7, 8], and gastric ulcers *in vivo* [9–13]. Therefore, we aimed to detect *H. coralloides* bioactivity by sequencing its genome. As a result of its potential activity, investigation of its genome may be necessary.

The recent advent of next-generation sequencing (NGS) technologies has facilitated genome sequencing and *de novo* assembly and increased the efficiency of genetic studies that have become a relative routine for genome analysis [14–16]. We previously spliced the *H. coralloides* genome by single-molecule real-time (SMRT) sequencing using individual polymerase molecules and the PacBio RS sequencing platform [17–19].

Here, we isolated mycelia from the fruiting bodies of *H. coralloides*, samples of which were collected from Lulang town, and enriched them by liquid fermentation. We assessed the overall profiles of the genes of *H. coralloides* and annotated their functions and obtained information about nonprotein coding RNAs (ncRNAs). We then investigated the underlying genetic basis of *H. coralloides*, by using the PacBio RS II, Illumina MiSeq, and Illumina NextSeq500 platforms in combination to sequence the *H. coralloides* genome and assemble it *de novo*. The results of the present study deepen the understanding of the *H. coralloides* genome and provide a basis for future studies of the genetic mechanisms that underlie the biological activities and potential medicinal value of this species. The genome dataset provides invaluable information about *H. coralloides* genes and their potential functional metabolites.

## 2. Materials and Methods

### 2.1. Sample Collection and Culture

Fruiting bodies of *H. coralloides* collected from Lulang town in Nyingchi City, Tibet Autonomous Region, China (29°5610.4^″^ N, 94°472.65^″^ E) on August 12, 2014, were morphologically identified and further characterized by sequencing the DNA of the internal transcribed spacer (ITS) region. Then, the mycelium of *H. coralloides* was isolated and purified from the fruiting body named *H. coralloides* tvtc0002 using PDA medium (potato dextrose agar medium, (w/v) 1% potato extract powder, 2% dextrose, and 1.5% agar, pH 6.5) and incubating at 25°C for 14 days. We then enlarged the mycelia by liquid fermentation in enriched PD medium (w/v; 1% potato extract powder, 2% dextrose, 0.1% MgSO_4_, 1% peptone, 0.5% beef extract powder, and 0.01% vitamin B_1_; pH 6.5) at 25°C and 200 rpm for 14 days.

### 2.2. DNA and RNA Isolation

The concentration, quality, and integrity of genomic DNA extracted using the cetyltrimethyl ammonium bromide (CTAB) method with minor modifications were determined using a Qubit Fluorometer (Invitrogen, USA) and a NanoDrop spectrophotometer (Thermo Scientific, USA). Sequencing libraries were generated using the TruSeq DNA Sample Preparation (Illumina, USA) and Template Prep Kits (Pacific Biosciences, USA).

The concentration, quality, and integrity of the total RNA isolated were determined using the TRIzol reagent (Invitrogen Life Technologies) and a NanoDrop spectrophotometer (Thermo Scientific). Three micrograms of the total RNA was used as input for the RNA sample preparation. Sequencing libraries were generated using the TruSeq RNA Sample Preparation Kit (Illumina, San Diego, CA, USA). Briefly, the mRNA was purified from total RNA using poly T oligo-attached magnetic beads. Fragmentation was carried out using divalent cations at elevated temperatures in an Illumina proprietary fragmentation buffer. First-strand cDNA was synthesized using random oligonucleotides and SuperScript II; then, second-strand cDNA was synthesized using DNA Polymerase I and RNase H. Remaining overhangs were converted into blunt ends using exonuclease/polymerase, after which they were removed. The 3′ends of the DNA fragments were adenylated and then ligated with PE adapter oligonucleotides to prepare for hybridization. To select 200 bp cDNA fragments, the library fragments were purified using the AMPure XP system (Beckman Coulter, Beverly, CA, USA). Thereafter, the DNA fragments with adaptor molecules ligated to both ends were selectively enriched using the Illumina PCR Primer Cocktail in a 15-cycle PCR reaction. The products were purified using the AMPure XP system and quantified using Agilent High Sensitivity DNA assay and a Bioanalyzer 2100 system (Agilent). The library was sequenced on a HiSeq platform (Illumina) at Shanghai Personal Biotechnology Cp. Ltd.

### 2.3. PacBio 20 K DNA Library Construction

Samples (20 *μ*g) of DNA (OD_260/_OD_280_ ≈ 1.8) were sheared using a Covaris® g-TUBE® device (Covaris, USA), diluted to 200–300 ng/*μ*L in elution buffer, and centrifuged at 5,500 rpm (2029 g) for 2 min on a MiniSpin Plus (Eppendorf). We constructed SMRTbell libraries according to the Procedure & Checklist-20 kb Template Preparation using the BluePippin™ Size Selection protocol (http://files.pacb.com/Training/IntroductiontoSMRTbellTemplatePreparation/story_content/external_files/Introduction%20to%20SMRTbell%E2%84%A2%20Template%20Preparation.pdf). Briefly, the library was run on a BluePippin system (Sage Science, MA, USA) to select SMRTbell templates > 10 kb. Sequencing primers were annealed to the hairpins of the templates and bound with P5 sequencing polymerase and MagBeads (Pacific Biosciences, CA, USA). The libraries were sequenced on a PacBio RS II platform at Shanghai Personal Biotechnology Cp. Ltd.

### 2.4. PE400 DNA Library Construction

The sequencing libraries were generated using the Nextera XT DNA Library Prep Kit (Illumina Inc.) as described by the manufacturer. Briefly, unfragmented gDNA was cleaved, tagged with Illumina adapters, and amplified using a limited-cycle PCR program with a unique combination of i7 and i5 index primers. The PCR products were purified using AMPure XP beads (Beckman Coulter Inc.) to remove short library fragments and then normalized with Nextera XT Library Normalization Beads (Illumina Inc.). The libraries were then sequenced on a MiSeq platform at Shanghai Personal Biotechnology Cp. Ltd.

### 2.5. Data Quality Control

The raw reads were filtered based on quality using FastQC with default parameters (http://www.bioinformatics.babraham.ac.uk/projects/fastqc), and the Q20, Q30, and GC contents were determined. For the raw data from NGS, 3′-adapter contaminant and tag sequence were removed (AdapterRemoval, version 2) [20]. Furthermore, we used NextClip (version 1.3.1) to remove the tag sequence of the reads in the Nextera Long Mate Pair library [21]. After quality correction of all the reads using Quake (version 0.3) and setting the k-mer to 17 [22], the reads that were ≤50 bp were removed.

### 2.6. Survey Analysis

Counting the number of occurrences of every k-mer in a DNA sequence is a central subproblem in many applications, including genome assembly, error correction of sequencing reads, fast multiple sequence alignment, and repeat detection [21]. We estimated genome size and heterogeneity using a survey analysis based on the k-mer counting algorithm [23]. We estimated the size of the genome (*G*) as follows:

(1) $$\begin{array}{c} G=\frac{\left(N\times \left(L–K+1\right)–B\right)}{D}, \end{array}$$

where *N* is the number of reads, *L* is the average length of the reads, *K* is k-mer, *B* is the k-mer for low frequency, and *D* is the peak value in the k-mer distribution diagram.

### 2.7. Genome Assembly and Analysis

The Falcon software (https://github.com/PacificBiosciences/FALCON-integrate) [24] assembles long reads and is thus suitable for genome assembly in diploid organisms. We used Falcon to assemble reads obtained from third-generation single molecular sequencing and constructed a contig with default parameters. The assembly results were then corrected based on NGS data using the Pilon software [25]. Finally, GapCloser (http://soap.genomics.org.cn/soapdenovo.html) [26] was used at default parameters to fill the gaps within the scaffolds. We mainly optimized the parameter values of Falcon, including -B, -t, -e, and -h, to improve genome assembly.

### 2.8. Evaluation of Integrity and Continuity of Genome Assembly

An accurate set of genes is needed to learn about species-specific properties, train gene-finding programs, and validate automatic predictions. However, many new genome projects lack comprehensive experimental data to derive a reliable initial set of genes. The amino acid sequences of a specific set of proteins are highly conserved over a wide range of eukaryotes. Therefore, comparing the assembled sequences with these proteins can determine the integrity of their sequences, which can then be used to indirectly evaluate the integrity and continuity of the assembled genome. In this study, we used Benchmarking Universal Single-Copy Orthologs (BUSCOs, http://busco.ezlab.org, v3.0.2) [27] to ensure the reliability of the assembled genome. BUSCO defined 290 conserved protein sequences and assumed that the sequences were present in all fungi. In the genome of *H. collaroides*, 283 conserved protein sequences were detected by BUSCO, accounting for 95.16% of the total conserved protein sequences. Among these, 238 genes were complete and single-copy BUSCOs, accounting for 82.1% of the total conserved protein sequences.

### 2.9. Sequence Alignment Analysis

High-quality, filtered data were aligned to the genome obtained from assembly using Burrows-Wheeler Aligner (BWA) (v. 0.7.12-r1039) at default parameters [28]. Duplicates were removed using MarkDuplicates in the Picard package. High-quality sequencing and assembly were second and third generations, respectively. The mapping sequence reached 92.46%, indicating that the results of the third-generation assembly could be analyzed.

### 2.10. Repeated Sequence Analysis

Repeated sequences are patterns of nucleic acids that occur in multiple copies throughout the genome. A significant fraction of genomic DNA is highly repetitive in many organisms [29]. Repeated sequences of nucleic acids occur in multiple copies throughout the genome and have been recognized as potential sources of genetic variation and regulation. We analyzed such sequences by homologous annotation using RepeatMasker v. 4.0.5 [30] based on the Repbase database [31] and by *de novo* annotation using RepeatModeler v. 1.0.4 (http://repeatmasker.org/RepeatModeler.html) based on the output files of RECON v.1.0.8 (http://selab.janelia.org/recon.html) and RepeatScout v. 1.0.5, http://repeatscout.bioprojects.org/).

### 2.11. Prediction of ncRNAs

Most genomes are apparently transcribed into ncRNAs [32] that are involved in many cellular processes, such as translation, RNA splicing, DNA replication, and gene regulation. Abundant and functionally important types of ncRNAs include transfer (tRNAs) and ribosomal RNAs (rRNAs) as well as small RNAs, such as microRNAs, siRNAs, snoRNAs, snRNAs, exRNAs, and the long ncRNAs. We predicted tRNA and rRNA using tRNAscan-SE v. 1.3.1 [33] and RNAmmer v.1.2 [34], respectively. Other types of ncRNAs were predicted by comparison with Rfam [35].

### 2.12. Prediction of Genes Encoding Proteins

We calculated the accuracy of gene prediction in eukaryotic genomic sequences as follows: (1) gene model was predicted *de novo* using Augustus v. 3.03 (http://augustus.gobics.de/submission) [36], glimmerHMM v.3.0.1 [37], and SNAP v. 2006-07-28 [38]; (2) homology was predicted using Exonerate v.2.2.0 (http://www.ebi.ac.uk/about/vertebrate-genomics/software/); (3) transcriptome was assembled *de novo* from RNA-Seq data using Trinity (v. r20140717) [39] and aligned using Program to Assemble Spliced Alignments (PASA) (v. r20140417) [40]; (4) the results of *de novo*, homologous, and RNA-Seq transcriptome predictions were integrated using EvidenceModeler v. r2012-06-25 with default parameters [40]. Boundaries of the predicted gene models were finally improved using PASA [41].

### 2.13. Functional Annotation

Based on the similarity of amino acid sequences in the protein domain, carbohydrate-active enzymes (CAZymes) are categorized as glycoside hydrolases (GHs), glycosyl transferases (GTs), polysaccharide lyases (PLs), carbohydrate esterases (CEs), carbohydrate-binding modules (CBMs), and auxiliary activities (AAs). We used HMMER (version 3.0) to predict CAZyme genes in the genome.

Genes encoding proteins were annotated using Gene Ontology (GO; http://www.geneontology.org), evolutionary genealogy of genes: Non-supervised Orthologous Groups (eggNOG; http://eggnog.embl.de), the Kyoto Encyclopedia of Genes and Genomes (KEGG; http://www.genome.ad.jp/kegg/), and Swiss-Prot (http://www.expasy.org/sprot/). Genes were also annotated by GO using BLAST2GO (http://www.blast2go.org/) [42] and eggNOG using BLASTP (BLAST version 2.2.28+; http://blast.ncbi.nlm.nih.gov/.cgi); KEGG orthology and pathways were annotated using the KEGG Automatic Annotation Server (KAAS; http://www.genome.jp/kegg/kaas/) [43].

### 2.14. Phylogeny Construction

The protein sequences of another 39 fungal species with available genome sequences were downloaded from the NCBI GenBank database (Table S1). Orthologous genes of *H. coralloides* and other fungal species were obtained using OrthoFinder v. 2.5.4 with the parameters -M dendroblast, -S blast, -A mafft, -T fasttree, and -l 1.5. Core single-copy orthologs were selected for subsequent phylogenetic analyses. The amino acid sequences of single-copy orthogroups were aligned using Muscle version 3.8.31; then, the best-aligned conserved blocks were extracted using Gblocks v. 0.91b at default parameters. A phylogenetic tree was constructed from the concatenated alignment by the neighbor-joining method using MEGAX v.10.2.6 with the p-distance model and 1,000 bootstraps. *Cantharellus anzutake* was set as the outgroup.

## 3. Results

We used the PacBio RS II, Illumina MiSeq, and Illumina NextSeq500 sequencing platforms in combination to sequence and assemble the genome of *H. coralloides de novo*.  Table 1 shows 307 contigs of 5,59,05,675 bp length.  Table 2 shows 11,736 genes and 73,583 total exons with lengths of 2,52,64,974 and 2,02,04,958 bp, respectively.

A total of 6,94,777, 3,33,64,830, and 3,40,63,012 reads were obtained using the PacBio 20 K DNA, PE400_DNA, and PE400_RNA libraries, respectively. The ratios (%) of GC for the three libraries were 50.28%, 53.65%, and 55.68%, respectively. The Q20 and Q30 values for the PE400_DNA and PE400_RNA libraries were 91.03% and 95.45% and 80.58% and 91.49%, respectively.

**Figure 1:**
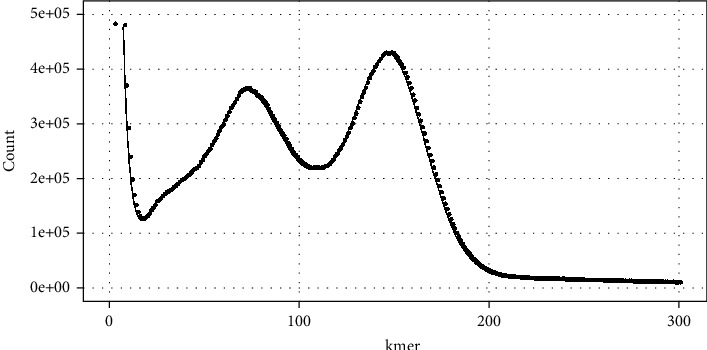
Distribution of 17-mers in *H. coralloides* genome. *X*-axis is 17-mer deep (*X*); *Y*-axis is the number of sequencing reads at that depth.

Analysis of the sequencing data with 17-mers revealed a genome of length 43.99 Mbp. The extent of heterozygosity was 0.847% (Table 3). In general, the k-mer value is usually set to 17, because the 17^th^ iteration of the 4 bases (ATCG; 4^17^) will reach 17G, which is enough to cover the whole genome, whereas k-mer of 15 will only result in 1G, which is insufficient to cover the whole genome. In general, a larger k-mer value is associated with a higher error ratio. We avoided palindromic sequences in k-mer analysis by setting odd k-mer values. Therefore, we set k-mer to 17, and Figure 1 shows a distribution map.

After genome assembly, 307 contigs were obtained, and the genome was 5,59,05,675 bp long. The minimum and maximum lengths of sequences were 4,325 and 22,71,665 bp, respectively, and the ratio of GC was 53.84%. The assembly results of the contig and scaffold were evaluated using Falcon, Pilon, and GapCloser, and the new genome data can be found online at NCBI (Assembly: ASM367540v1).

BUSCO was used to define 290 conserved protein sequences and assumed that they were common to 85 fungal species. We determined that 283 conserved protein sequences accounted for 97.6% of the total number of conserved protein sequences. Among these, 238 were complete and single-copy BUSCOs, accounting for 82.1% of the total. These results indicated good integrity and continuity of the genome assembly (Table 4). After sequence alignment, 2,95,13,883 reads were mapped to the genome at a rate of 92.46% and an average sequencing depth of 114.6. The coverage ≥ 4, ≥10, and ≥20 were 96.7%, 95.93%, and 94.35%.

**Figure 2:**
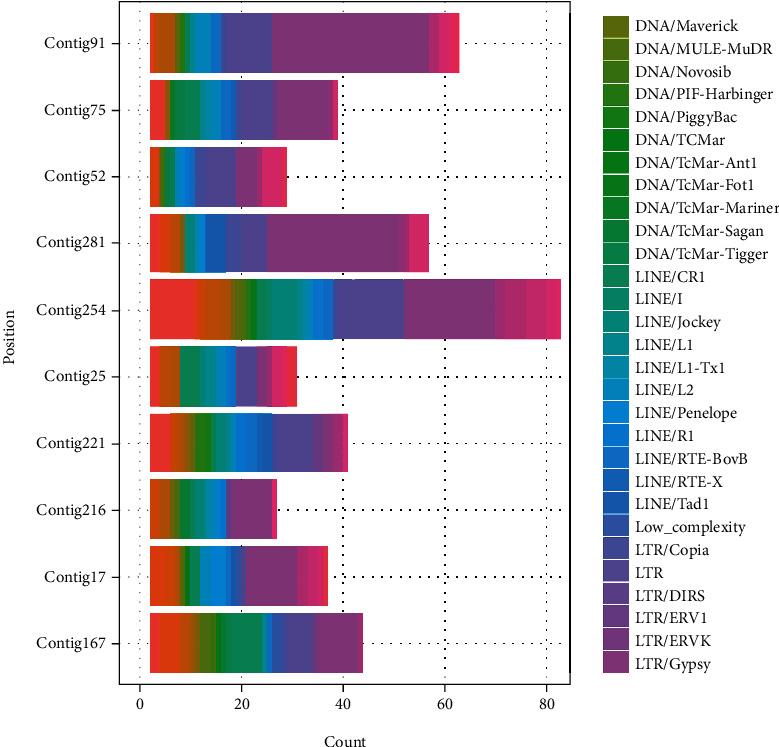
Composition of repeated sequences in each chromosome in the genome. Title is the class and family of repetitive DNA shown as DNA TIRs (terminal inverted repeats), LINEs (long interspersed nuclear elements), and LTRs (long terminal repeat). The family of TIRs of *H. coralloides* are shown as Maverick (virus-like DNA transposon), MULE-MuDR (Mutator-like transposable elements-MuDR transposon), Novosib (Novosib transposon), PIF-Harbinger (PIF-Harbinger transposon), PiggyBac (PiggyBac transposon), and TcMar (TcMar transposon).

Table 5 shows the results of the repeated sequence analysis. We identified 2,525 repeated sequences, including 2,353 interspersed repeats, 33 satellites, 121 simple repeats, and 18 low complexities. The interspersed repeats included 1,827 retroelements, 466 DNA transposons, and 60 unclassified repeats (Figure 2).

The copy numbers of ncRNAs, rRNAs, tRNAs, snoRNAs, snRNAs, and other ncRNAs were 9, 270, 6, 21, and 32, respectively.  Table 6 shows the average and total lengths of ncRNAs, as well as the proportion of ncRNA accounting for the genome. In total, 11,736 genes encoding proteins were predicted, with a total length of 2,52,64,974 bp. The average length of the genes encoding proteins was 2,152 bp.

**Figure 3:**
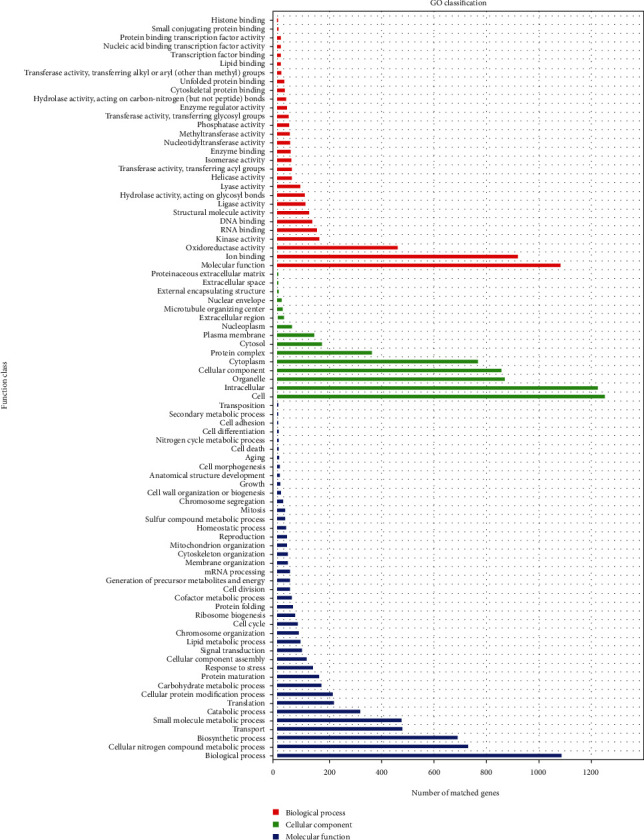
Gene Ontology (GO) functional annotation of genome. Blue, biological process; red, molecular function; green, cellular component. *X*-axis, number of matched genes; *Y*-axis (right), function class.

We assigned 539 H*. coralloides* genes to CAZyme families, as defined in the CAZy database. The results of CAZyme analysis predicted that 152 genes had auxiliary activities, 19 were carbohydrate-binding modules, 83 were CEs, 210 were GHs, and 74 were GTs. The 3,675 annotated genes in the GO database were associated with biological processes (BPs), cellular components (CCs), and molecular function (MF) terms. In detail, 41 BP aspects were annotated, including biological, cellular nitrogen compound metabolic, and biological processes. A total of 15 CC aspects were identified, including cell, intracellular, and organelle. Additionally, 29 MF terms were annotated, including molecular function, ion binding, and oxidoreductase activity (Figure 3).

**Figure 4:**
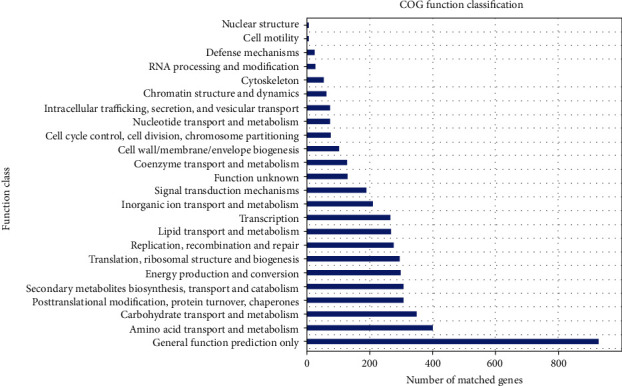
Clusters of orthologous groups (COG) of protein annotations of genome. *X*-axis, COG categories; *Y*-axis, number of matched genes. Total number of genes enriched in KEGG pathways was 1,311.

**Figure 5:**
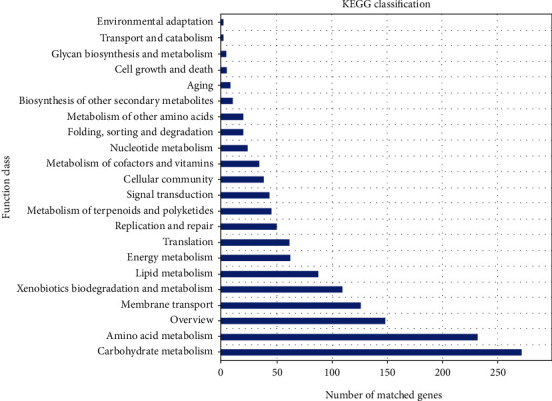
Kyoto Encyclopedia of Genes and Genomes (KEGG) annotation of genome. *X*-axis, number of matched genes; *Y*-axis (right), function class.

The comparison of gene sets with the evolutionary eggNOG database using BLASTP (BLAST v. 2.2.28+; http://blast.ncbi.nlm.nih.gov/Blast.cgi) resulted in 3,602 annotated genes and clusters of orthologous groups (COG) of proteins.  Figure 4 shows that 7.92%, 3.39%, and 2.96% of genes encoding proteins, respectively, were classified into function R (general function prediction only), function E (amino acid transport and metabolism), and function G (carbohydrate transport and metabolism). The pathways associated with metabolism, namely, carbohydrate and amino acid metabolism as well as xenobiotic biodegradation and metabolism, were associated with environmental information processing, such as membrane transport and signal transduction (Figure 5).

**Figure 6:**
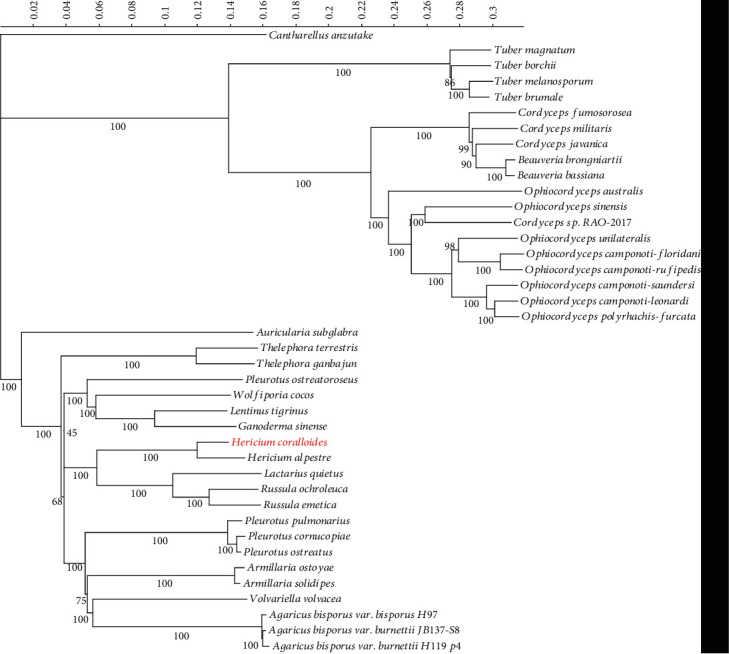
The phylogenetic tree based on single-copy ortholog genes between *H. coralloides* and 39 other fungal species.

We identified 169 single-copy orthogroups from *H. coralloides* and other fungal species and used corresponding amino acid sequences to construct a phylogenetic tree (Figure 6) in which *H. coralloides* was clustered with *Hericium alpestre*, which is another species of the same genus. *Russula* and *Lactarius* were the most closely related genera to *Hericium*, followed by *Pleurotus*, *Wolfiporia*, *Lentinus*, and *Ganoderma*.

## 4. Discussion

In this study, the *H. coralloides* genome was sequenced and assembled *de novo* for the first time using PacBio RS II, Illumina MiSeq, and Illumina NextSeq500 platforms. The assembled genome was 5,59,05,675 bp in length and included 307 contigs. The mapping rate of reads obtained from PE400 DNA in the assembled genome was 92.46%. We identified 2,525 repeated sequences of 14,23,274 bp. We also predicted 48,895 bp ncRNAs and 25,264,974 genes encoding proteins that were annotated in the GO, eggNOG, and KEGG databases.

A significant fraction of genomic DNA is highly repetitive in many organisms. A growing body of literature suggests that such sequences are vital to the genome [44]. The major categories of repeated sequences are terminal, tandem, and interspersed repeats. We identified 2,525 repeated sequences, most (2,353) of which were interspersed. All eukaryotic genomes have interspersed repeats, which are distributed throughout the genome and are not adjacent to each other. The repeated sequences vary depending on the organism and other factors [45]. Interspersed repeats comprise an isolating mechanism that enables new genes to evolve without interference from the progenitor gene. Therefore, repetitive sequence analysis might help to understand the evolution of *H. coralloides*.

The word “gene” has been synonymous for several decades with a genome encoding mRNAs that are translated into proteins. However, recent genome-wide studies have revealed thousands of regulatory ncRNAs that produce a functional RNA product instead of a translated protein [46]. The range and importance of such genes have only recently become apparent, with known ncRNAs playing a wide range of intracellular structural, regulatory, and catalytic roles [35, 47]. Our findings were similar to those of another study on the *Cordyceps guangdongensis* genome, which contains 314 ncRNAs. We predicted 338 ncRNAs among which, 270 were tRNAs that are key components of the translational machinery connecting the genetic code with the amino acid sequences of proteins. They comprise up to 15% of the total cellular RNA and are among the most abundant cellular transcripts [48]. Furthermore, tRNAs regulate numerous cellular and metabolic processes in eukaryotes and prokaryotes. The prediction of ncRNA in *H. coralloides* might contribute to further exploration of its cellular and metabolic processes.

CAZymes build and break down complex carbohydrates and glycoconjugates for many biological roles [49]. A saprophytic lifestyle is closely associated with CAZymes in fungal genomes [50, 51]. *H. coralloides* is a species of wood-rotting fungi, suggesting that it has ligninolytic, cellulolytic, hemicellulolytic, and pectinolytic properties that could be further investigated based on the annotations of CAZymes for some industrial applications. The annotation of CAZymes of *H. coralloides* led to the identification of increased levels of GHs and AAs and decreased levels of CBMs, which might provide a deeper understanding of the ecological roles and carbohydrate metabolic mechanisms of *H. coralloides*. In addition, *H. coralloides* laccase has various substrates, the most sensitive of which is 2,2′-azino-bis(3-ethylbenzothiazoline-6-sulfonic acid) [52]. Furthermore, *H. coralloides* produces an extracellular laccase, which catalyzes epitheaflagallin 3-O-gallate (ETFGg) synthesis from epigallocatechin gallate (EGCg) with gallic acid [11]. To the best of our knowledge, ETFGg has physiological functions. Therefore, more genes encoding useful enzymes might be discovered based on whole-genome sequencing.

Genomic sequencing has suggested that most genes that specify core biological functions are shared by all eukaryotes [53]. Rational annotation of proteins that are encoded in the sequenced genome can render genome sequences useful for functional and evolutionary investigations [54]. Therefore, we studied a series of functional annotations for the genome and identified numerous metabolic and organismal system-associated functions and pathways, such as cellular nitrogen compound metabolic process and carbohydrate metabolism. Because *H. coralloides* has an abundance of compounds from which metabolites can be extracted using biochemical and physiological means, the present findings serve as an important platform for further biological investigation into the microbe. The positive regulation of these functions might explain the edible and medicinal properties of *H. coralloides*. We also found that *H. coralloides* is a bioactive repository of natural compounds as the extracts of mycelia and fruiting bodies contained many metabolites with neurotrophic effects [55] and antioxidant activity [56], such as corallocins A-C [57], spirobenzofuran [58], and other new compounds [56]. The edible and medicinal properties of *H. coralloides* are further supported by phylogenetic findings indicating its close phylogenetic proximity to the edible fungi genera, *Russula* and *Lactarius*, which have high nutritional value [59].

In summary, we generated and analyzed a draft genome assembly of *H. coralloides* using a combined SMRT long-read sequencing approach. Our novel genomic data will provide valuable resources for edible and medical mushroom investigation and facilitate further studies on the genetic basis of *H. coralloides*. Our findings might also help further explorations on the bioactivity and evolution of *H. coralloides*.

## 5. Conclusions

The study sequenced the entire genome of *H. coralloides* (NCBI; Assembly: ASM367540v1), which is widely applied in TCM. The assembled genome was 5,59,05,675 bp in length and had 307 contigs. We identified 11,736 genes and 73,583 exons of lengths 2,52,64,974 and 2,02,04,958 bp, respectively. The mapping rate of the reads obtained from PE400 DNA in the assembled genome was 92.46%. We also predicted 48,895 bp ncRNAs and 11,736 genes encoding proteins that were annotated in the GO, eggNOG, and KEGG databases. In the future, we plan to detect the bioactive genes in *H. coralloides* and their related processes using genomic data.

## Figures and Tables

**Table 1:** Statistics for the final assemblies of the *H. coralloides* genome.

Property	Contig
Total sequence Num.	307
Total sequence length (bp)	55,905,675
Min. sequence length (bp)	4,325
Max. sequence length (bp)	2,271,665
N50	441,150
GC (%)	53.84
N Num.	0
N rate (%)	0

**Table 2:** Prediction of genes in the assembled *H. coralloides* genome.

Property	Value
Total gene length (bp)	25,264,974
Total gene Num.	11,736
Average gene length (bp)	2,152
Gene percentage of genome (%)	45.19
Total exon length (bp)	20,204,958
Total exon Num.	73,583
Average exon length (bp)	274
Average exons per gene	6.2
Exon percentage of genome %	36.14
Total CDS length (bp)	16,343,018
Average CDS length (bp)	1,392
CDS percentage of genome %	29.23
Average intron length (bp)	76.6

**Table 3:** Result of 17-mer analysis.

Property	Value
k-mer	17
k-mer Num.	55,483,329
k-mer peak (×)	148
Low frequency k-mer Num. (≤2)	1,340,810
Avg. read length (bp)	220
Total read Num.	31,921,174
Heterozygosity (%)	0.847
Repetitive 17-mer fraction	0.026
Genome size (bp)	43,990,397

k-mer: sequence of k bases; k-mer Num.: number of k-mer; k-mer peak (×): peak value of k-mer; low frequency k-mer Num. (≤2): k-mer of low-frequency; Avg. read length (bp): average length of reads; total read Num.: total number of reads; repetitive 17-mer fraction: proportion of repeated sequence.

**Table 4:** Evaluation of integrity and continuity of genome assembly.

Property	Number	Percent (%)
Complete BUSCOs	283	97.6
Complete and single-copy BUSCOs	238	82.1
Complete and duplicated BUSCOs	45	15.5
Fragmented BUSCOs	1	0.3
Missing BUSCOs	6	2.1
Total BUSCO groups searched	290	100

Total BUSCO groups searched: the single-copy direct line homologous gene database used by BUSCO is fungi_odb9, which provides 290 single-copy genes from 85 fungi.

**Table 5:** Statistical result of repeated sequences.

Elements	Number of elements	Length (bp)	Percentage of genome
Interspersed repeats	2,353	1,389,268	2.49
Retroelements	1,827	1,303,927	2.33
DNA transposons	466	71,198	0.13
Unclassified	60	14,143	0.03
Satellites	33	9,162	0.02
Simple repeats	121	20,741	0.04
Low complexity	18	4,103	0.01
Summary	2,525	1,423,274	3.00

**Table 6:** Results of noncoding RNA (ncRNA) prediction.

Type	Copy	Avg. length (bp)	Total length (bp)	% of genome
rRNA	9	1,923	17,309	0.030961
tRNA	270	88	23,845	0.042652
snoRNA	6	99	594	0.001062
CD-box	6	99	594	0.001062
HACA-box	-	-	-	-
scaRNA	-	-	-	-
snRNA	21	130	2733	0.004888
Other ncRNA	32	137	4414	0.007895
Summary	338	-	48,895	0.087458

ncRNA type: type of ncRNA; copy: copy number of ncRNA; Avg. length (bp): average length of ncRNA; total length (bp): total length of ncRNA; % of genome: percentage of ncRNA in genome.

## Data Availability

Genomic data can be obtained from NCBI (https://www.ncbi.nlm.nih.gov/) (Assembly: ASM367540v1).

## Supplementary Materials

Supplementary Materials Table S1: the list of fungi species used for phylogenic construction in this study.
