# Supplementary material for: Genome Sequencing of Hericium coralloides by a Combination of PacBio RS II and Next-Generation Sequencing Platforms
Source: Int J Genomics. 2022 Jan 31;2022:4017654. doi: 10.1155/2022/4017654 (PMC8820905; doi:10.1155/2022/4017654)
Supplement: Supplementary Materials — Table S1: the list of fungi species used for phylogenic construction in this study. [file 4017654.f1.docx]

| Table S1. The list of fungi species used for phylogenic construction in this study. | | |
| --- | --- | --- |
| Genus | Species | Genome accession No. |
| *Agaricus* | *Agaricus bisporus var. bisporus H97* | GCA_000300575.1 |
| *Agaricus* | *Agaricus bisporus var. burnettii* | GCA_014872705.1 |
| *Agaricus* | *Agaricus bisporus var. burnettii JB137-S8* | GCA_000300555.1 |
| *Armillaria* | *Armillaria solidipes* | GCA_002307675.1 |
| *Armillaria* | *Armillaria ostoyae* | GCA_900157425.1 |
| *Auricularia* | *Auricularia subglabra* | GCA_000265015.1 |
| *Cantharellus* | *Cantharellus anzutake* | GCA_015039405.1 |
| *Cordyceps* | *Cordyceps militaris* | GCA_000225605.1 |
| *Cordyceps* | *Cordyceps fumosorosea* | GCA_001636725.1 |
| *Cordyceps* | *Cordyceps javanica* | GCA_006981975.1 |
| *Cordyceps* | *Beauveria bassiana* | GCA_000280675.1 |
| *Cordyceps* | *Ophiocordyceps sinensis* | GCA_012934285.1 |
| *Cordyceps* | *Beauveria brongniartii* | GCA_001636735.1 |
| *Ganoderma* | *Ganoderma sinense* | GCA_002760635.1 |
| *Hericium* | *Hericium alpestre* | GCA_004681135.1 |
| *Lactarius* | *Lactarius quietus* | GCA_015025155.1 |
| *Lentinus* | *Lentinus tigrinus* | GCA_003813185.1 |
| *Ophiocordyceps* | *Ophiocordyceps polyrhachis-furcata* | GCA_001633055.2 |
| *Ophiocordyceps* | *Ophiocordyceps sinensis* | GCA_012934285.1 |
| *Ophiocordyceps* | *Ophiocordyceps camponoti-floridani* | GCA_012980515.1 |
| *Ophiocordyceps* | *Ophiocordyceps camponoti-saundersi (nom. inval.)* | GCA_003339415.1 |
| *Ophiocordyceps* | *Ophiocordyceps camponoti-leonardi (nom. inval.)* | GCA_003339455.1 |
| *Ophiocordyceps* | *Ophiocordyceps camponoti-rufipedis* | GCA_002591395.1 |
| *Ophiocordyceps* | *Ophiocordyceps australis* | GCA_002591415.1 |
| *Ophiocordyceps* | *Ophiocordyceps unilateralis* | GCA_001272575.2 |
| *Pleurotus* | *Pleurotus ostreatus* | GCA_014466165.1 |
| *Pleurotus* | *Pleurotus cornucopiae* | GCA_019677325.2 |
| *Pleurotus* | *Pleurotus pulmonarius* | GCA_012980535.1 |
| *Pleurotus* | *Pleurotus ostreatoroseus* | GCA_005298045.1 |
| *Russula* | *Russula ochroleuca* | GCA_015178965.1 |
| *Russula* | *Russula emetica* | GCA_015178925.1 |
| *Thelephora* | *Thelephora terrestris* | GCA_015956445.1 |
| *Thelephora* | *Thelephora ganbajun* | GCA_014904855.1 |
| *Tuber* | *Tuber melanosporum* | GCA_000151645.1 |
| *Tuber* | *Tuber borchii* | GCA_003070745.1 |
| *Tuber* | *Tuber magnatum* | GCA_003182015.1 |
| *Tuber* | *Tuber brumale* | GCA_014065205.1 |
| *Volvariella* | *Volvariella volvacea* | GCA_001691835.3 |
| *wolfiporia* | *Wolfiporia cocos* | GCA_000344635.1 |
